# Neurophysiological Evidence for a Compensatory Activity during a Simple Oddball Task in Adolescents with Type 1 Diabetes Mellitus

**DOI:** 10.1155/2018/8105407

**Published:** 2018-07-08

**Authors:** Tereza Vitvarová, David Neumann, Radka Šimáková, Jan Kremláček

**Affiliations:** ^1^Department of Pediatrics, University Hospital Hradec Kralove, Faculty of Medicine in Hradec Kralove, Charles University, Hradec Kralove, Czech Republic; ^2^Philosophy Faculty, Palacky University Olomouc, Olomouc, Czech Republic; ^3^Department of Pathological Physiology, Faculty of Medicine in Hradec Kralove, Charles University, Hradec Kralove, Czech Republic

## Abstract

**Objective:**

The poor metabolic control in type 1 diabetes mellitus (T1D) has a negative impact on the developing brain. Hyperglycemia and glycemic fluctuations disrupt mainly executive functions. To assess a hypothesized deficit of the executive functions, we evaluated visual processing and reaction time in an oddball task.

**Methods:**

Oddball visual event-related potentials (ERPs), reaction time, and pattern-reversal visual evoked potentials (VEPs) were examined in a cohort of twenty-two 12- to 18-year-old T1D patients without diabetic retinopathy at normal glycemia and in nineteen 10- to 21-year-old healthy controls.

**Results:**

The P100 peak time of the VEPs was significantly prolonged in T1D patients compared with the control group (*p* < 0.017). In contrast to the deteriorated sensory response, the area under the curve of the P3b component of the ERPs was significantly larger (*p* = 0.035) in patients, while reaction time in the same task did not differ between groups (*p* = 0.713).

**Conclusions:**

The deterioration on a sensory level, enhanced activity during cognitive processing, and balanced behavioral response support the view that neuroplasticity counterbalances the neural impairment by enhanced cognitive processing to achieve normal behavioral performance in T1D adolescents.

## 1. Introduction

Executive functions (EFs) have a pivotal role in controlling adaptive behavior, activating motivation, and maintaining or changing the direction of action, thereby influencing various life activities, for example, school readiness and school success [1]. Altered EFs are thought to contribute to suboptimal adherence to treatment regimens in type 1 diabetes mellitus (T1D) [2–4], and neurodevelopmental problems are assumed to precede poor metabolic control [5].

Consequently, hypoglycemia and prolonged periods of hyperglycemia have a negative impact on the developing nervous system and can harm cognitive processes [6].

Such interrelated processes could send the patient's condition into a spiral of deterioration; therefore, an understanding of EFs in T1D is clinically important.

Various performance measures and rating inventories are widely used to assess EFs [7]. Aside from these neuropsychological approaches, objective neurophysiological methods offer noninvasive evaluation of the EFs via brain activity recording. The widely used oddball electrophysiological test presents two types of stimuli with different probabilities [8]. To succeed in the oddball task, one must follow the stream of stimuli for approximately 5 minutes, differentiate between rare and frequent stimuli, and respond selectively to the rare, or target, stimulus. On the neurobiological level, such behavior requires not only effective sensory and motor processing but also attention as part of inhibitory control, as well as working memory, both of which are core elements of executive functioning [1]. On a neural level, the response to the target stimuli evokes the P3b component of an event-related potential (ERP) [9, 10]. P3b originates from the activation of many regions of the neocortex and limbic system. It can be recorded noninvasively from the scalp as a positive potential with a maximum in the centroparietal area [11]. In the oddball test, a choice reaction time can also be recorded as a behavioral measure.

Adolescents with T1D constitute a risk group for suboptimal glycemic control. In the present study, we investigated whether the generally observed drop in therapy adherence in these patients is linked to a prolonged or delayed P3b component as an electrophysiological marker of EF impairment.

## 2. Methods

### 2.1. Study Design

In our observational case-control study, we compared a group of adolescents with T1D to an age- and gender-matched control group. The study was approved by the Ethical Committee of the University Hospital Hradec Kralove (numbers 201511 S10P and 201511S 2sP) and conducted according to the principles of the Declaration of Helsinki [12]. Patients were invited to join the study during regular visits to their diabetologist, and each invited patient was asked to bring a friend as a control subject. Attention-deficit/hyperactivity disorder, learning disorders, and retinopathy or other visual deficit were study exclusion criteria. After being informed about the study, all participants and their parents signed informed consent documents.

### 2.2. Participants, Diabetes, and Glycemic History

For the study, we recruited twenty-two adolescent patients with T1D, preferably with a known long-term history of poor compliance with therapy, older than eleven years of age, with disease duration over two years. All of them were frequently consulted on an outpatient and inpatient basis, alone, with parents, or in a group of peers, to improve their glycemic control. T1D duration, school performance (excellent, modest, and inferior), serious T1D complications (diabetic ketoacidosis with hospital admission, severe hypoglycemia, and long-lasting glycemic excursions), insulin regimen (human insulin, insulin analogs, and continuous subcutaneous insulin infusion), and mode of blood glucose monitoring were recorded, along with therapy-related measures of hemoglobin A1c (HbA1c) in a one-year period before testing and glycemia when tested.

### 2.3. HbA1c and Glycemia Assessment

Glycated hemoglobin was measured by an automated high-performance liquid chromatography gradient elution analyzer (Arkray Adams A1c HA-8180, Arkray Inc., Japan). Long-lasting glycemic variability was expressed as the coefficient of variability of HbA1c [13]. To determine mean and variability of the glycated hemoglobin, we used three or four measurements recorded over at least one year before the experiment. Actual glycemia just before the electrophysiological procedure was determined using personal glucose meters conforming to the ISO 15197:2013 standard. For post hoc evaluation, we calculated an exposure score as the sum of the *z*-transformed duration of T1D and glycemic control [14].

### 2.4. Electrophysiological Procedures

Landolt rings (EN ISO 8596) and the hole-in-the-card test determined subjects' visual acuity and ocular dominance, respectively. Subjects used their dominant eye in all tests.

Pathology in the sense of prolonged latency or decreased amplitude of P3b may be caused by an impairment of sensory processing. Since we used a visual oddball paradigm, we also evaluated visual acuity and recorded visual evoked potentials (VEPs) using equipment described below to assess sensory processing. In particular, we measured the pattern-reversal P100 peak, reflecting primary visual cortex activation [15].

ERP and VEP measurements were performed in a darkened, sound-attenuated, electromagnetically shielded room with a background luminance of 0.1 cd/m^2^. During experiments, the subjects sat in a comfortable dental chair with a neck support to reduce muscle artifacts. A near-infrared camera monitored correct fixation. All stimuli were presented on a 21^″^ computer monitor (Vision Master Pro 510, Iiyama, Japan) subtending 37° × 28° of the visual field from an observing distance of 0.6 m. Stimuli for VEPs were presented using the Visual Stimulus Generator 2/5 (CRS Ltd., UK) at a vertical refresh frequency of 105 Hz. The ERP stimuli were presented by Psychtoolbox-3 [16] at a vertical refresh frequency of 75 Hz. Recorded epochs were synchronized with a backward trace of the monitor's electron beam just before the first video frame of an appropriate stimulus change.

VEPs/ERPs were recorded from six unipolar derivations (O_Z_, P_Z_, C_Z_, F_Z_, and O_L_; O_R_—5 cm left and right from the O_Z_) with a right earlobe reference (A_2_). The minimum set of recording derivations was chosen on the basis of a previous topographical study concerning the scalp distribution of ERPs [9] and VEPs [17]. The ground electrode was connected to the reference. All electrode impedances were kept below 10 kΩ. The signal was amplified in the frequency band of 0.3–100 Hz (PSYLAB, System 5, Contact Precision Instruments, USA).

### 2.5. Cognitive Event-Related Potentials

ERPs were recorded during the visual oddball test in which the white letter X (frequent nontarget stimulus with a probability of 75%) and Arabic digits 1–9 (rare target stimuli with a probability of 25%) appeared pseudorandomly. The “X” or the digit, subtending 5.7° × 6.3°, was displayed for 500 ms in the center of the black stimulus field, followed by a blank screen with a fixation point displayed for 500 ms. The mean luminance was 1 cd/m^2^. The subjects were instructed to press a handheld button as soon as possible whenever a rare stimulus appeared. This arrangement enabled an evaluation of the latency and amplitude of the main ERP peak P3b and the reaction time. Subjects learned the experimental task in a short training session before the test.

Twenty poststimulus EEG epochs of 1000 ms after target stimuli and 20 randomly selected epochs after nontarget stimuli were sampled at 250 Hz frequency. Epochs with absolute amplitude exceeding 70 *μ*V were rejected. The rest of the responses were smoothed with a second-order polynomial Savitzky-Golay filter across 21 samples. The mean interpeak amplitudes (P3b − (N2 + N3)/2), the peak time of the P3b response, and the reaction times were evaluated offline.

### 2.6. Pattern-Reversal Visual Evoked Potentials

Forty reversals of a high-contrast black and white checkerboard pattern within 20 seconds evoked pattern-reversal VEPs. Two checkerboard stimulations with check sizes of 40 arcmin (PR-VEP 40′) and 20 arcmin (PR-VEP 20′) were used. The mean luminance of 17 cd/m^2^ stayed constant. The subjects were instructed to keep their gaze on the fixation point during the recording. EEG poststimulus epochs of 440 ms duration were sampled at 500 Hz. Epochs with absolute amplitudes larger than 70 *μ*V were rejected. The rest of the responses were smoothed with a second-order polynomial Savitzky-Golay filter across 21 samples. The number of samples was determined empirically to remove high-frequency noise. Both VEP variants were examined twice. The mean interpeak amplitudes (P100 − (N75 + N145)/2) and the peak time of P100 were evaluated offline.

### 2.7. Analysis and Statistics

Signal filtering, extraction of the parameters of interest, and data plotting were conducted in MATLAB Release 2017a (MathWorks, USA). Statistical analysis was performed with the “nortest” package in the software R 3.4.0 [18]. The normality of the data distribution was assessed by the Anderson-Darling test. To compare variables between groups, we used a Wilcoxon rank-sum test or a *t*-test for data with a nonnormal or normal distribution, respectively. The results are presented as the median and interquartile range. Relationships between continuous clinical, behavioral, and EF markers of interest were calculated using Pearson's correlation coefficient or Spearman's rank correlation, depending on the normality or nonnormality of the data distribution. Differences among groups defined by categorical variables (sex, insulin regimen, T1D complications, and school performance) were evaluated using ANOVA with post hoc *t*-tests corrected for multiple comparisons. Gender representation was compared between groups by Fisher's exact test. The level of statistical significance was preset to *p* < 0.05.

## 3. Results

Twenty-two adolescents with T1D (12 girls and 10 boys aged 12–18 years) were selected. The control group consisted of 19 age-matched healthy friends of patients (11 girls and 8 boys aged 10–21 years).  Table 1 lists characteristics of the study groups. Patients were treated on fixed insulin regimens with either insulin applicators (10 patients) or insulin pumps (12 patients). They used personal glucometers as their standard monitoring devices and were familiar with occasional measurement by glucose sensors. In the T1D group, electrophysiological testing was performed at a blood glucose level of 3.9 to 10.0 mmol/l. There was no apparent retinopathy within our group, which urges absence of microvascular changes [19].

**Figure 1:**
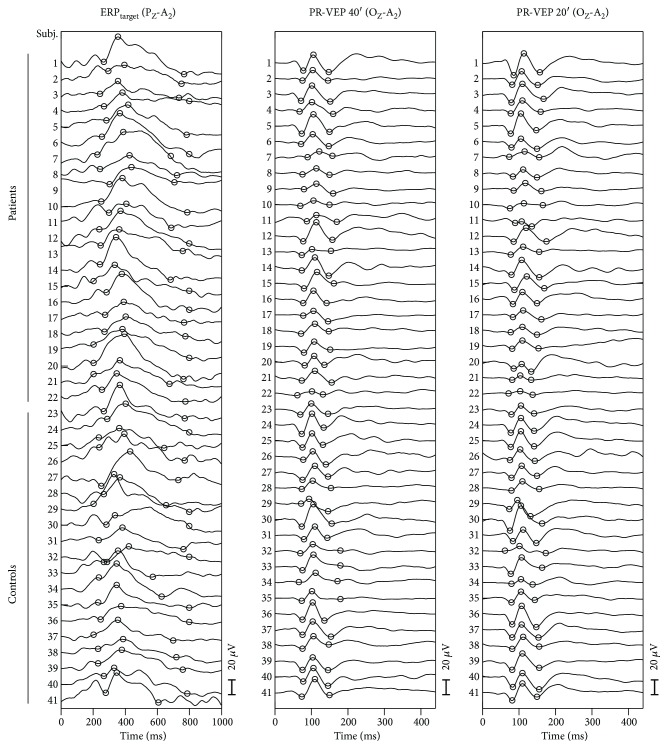
Individual ERP and VEP traces. Single subjects' ERPs and VEPs are plotted as thick or thin lines for patients and controls, respectively. The columns correspond to the selected derivation of examined ERP/VEPs, and the subjects' responses form the rows. The marked peaks were used for the statistical analysis. For the latency assessment, we used the middle marker; for amplitude, an average of two interpeak values (see Methods). There is an apparent increase in the area under curve of the P3b peak in the target ERP for patients, as we confirmed in the intergroup comparison (*p* = 0.035). Further, there is a slight but significant (*p* < 0.016) time shift of the middle marker of the pattern-reversal VEPs—the P100 peak—for both stimulation patterns (PR-VEP 40′ and 20′).

Brain reactions to stimuli in the oddball paradigm and other sensory responses were reliably recorded in all subjects (see Figure 1). There were no differences between the group of adolescents with T1D and the controls in age (*p* = 0.231), gender (*p* = 1.0), visual acuity (*p* = 0.217), or amplitudes of sensory responses (*p* > 0.066) (see Table 2). Nevertheless, in the T1D group, we found a significant delay in the P100 peak time in PR-VEP 40′ and PR-VEP 20′ (T1D subjects (median, interquartile range): 107, 105–113 ms; 110, 108–114 ms, resp.; controls: 104, 103–106 ms; 108, 106–110 ms, resp.); *p* = 0.017 and *p* = 0.012.

In the P3b component, neither the peak time difference of 16 ms between groups (T1D group: 384, 365–396 ms; controls: 368, 348–386 ms) (*p* = 0.181) nor the difference in P3b amplitude at the peak maximum (T1D subjects: 27.1, 21.2–33.5 *μ*V; controls: 23.6, 19.7–28.8 *μ*V) (*p* = 0.331) were significant.

**Figure 2:**
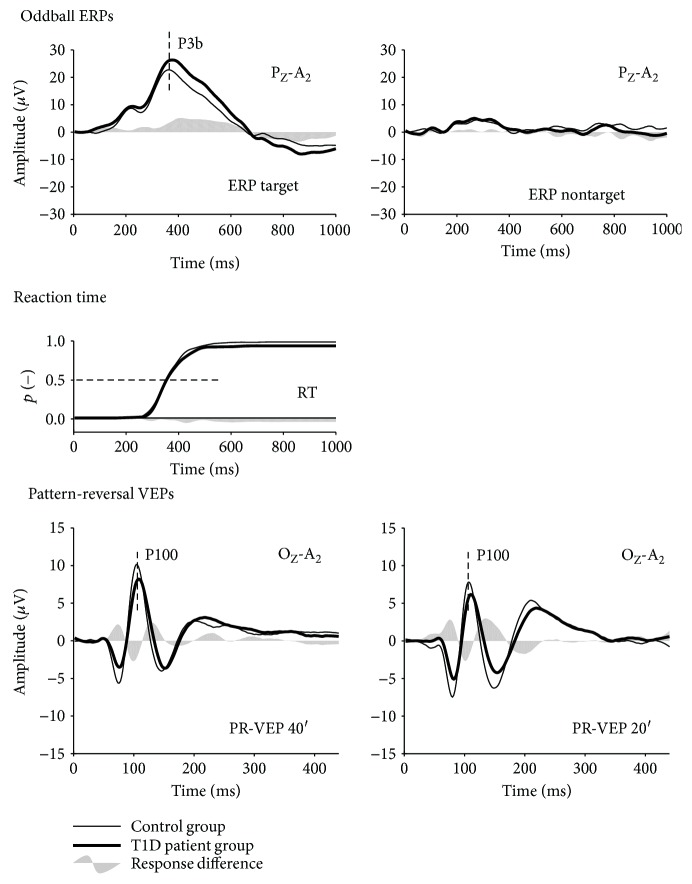
ERP and VEP grand averages in patients and controls. The patient and control grand average ERPs are plotted as the thick and thin lines, respectively; the between-group ERP difference is the gray area in the whole figure. The two horizontal gray lines show the level of variability, pointwise calculated as the mean plus 2.5 times the standard deviation of values in the first 60 ms for ERPs and 30 ms for VEPs. The first row depicts ERPs to target (left plot) and nontarget (right plot) stimuli in the oddball cognitive task, evaluated from the parietal derivation. The second row shows the cumulative distribution function of pressing the response button—the reaction time in response to the target stimulus. The pattern-reversal VEP PR-VEP 40′ (left plot) and PR-VEP 20′ (right plot), recorded in the central occipital derivation, are shown in the third row; motion-onset VEPs from the periphery (M-VEP 20°, left plot) and the central visual field (M-VEP C8°, right plot), recorded from the parietal derivation, are shown in the fourth row.

However, the increase in P3b amplitude in the T1D group became prominent after the peak maximum (see Figure 2). The area under the curve (AUC) for the interval from 360 to 500 ms was significantly larger in the T1D group (2994; 2541–4047 *μ*V ·ms) than in the control group (2446; 1838–2987 *μ*V ·ms), *p* = 0.035.

In clinical diagnostics, a significant difference between groups is not directly usable; therefore, we assessed the discriminative potential of statistically significant parameters by an ROC (receiver operating characteristic) analysis [20]. Each threshold was determined as the point of maximal sensitivity and specificity [21]. The thresholds were as follows: P3b AUC, 2487 *μ*V ms; P100 peak in PR-VEP 40′, 105 ms; and P100 peak in PR-VEP 20′, 109 ms. For those thresholds, the ROCs showed sensitivity of 53, 58, and 68%, respectively, and specificity of 77, 73, and 64%, respectively. The control group was used to define a set of high-specificity (99.8%) reference limits. For P3b AUC, PR-VEP 40′ P100 latency, and PR-VEP 20′ P100 latency, respectively, 27% (6 patients), 27% (6), and 41% (9) of patients were outside these reference limits.

On the behavioral level, neither the reaction times (T1D subjects: 354, 333–384 ms; controls: 348, 332–372 ms), *p* = 0.714, nor the accuracy of the discrimination (median for both groups was 100%), *p* = 0.24, differed.

## 4. Post Hoc Analysis of EFs in T1D Subjects

### 4.1. Age of Onset and Duration of Diabetes

Subjects with T1D onset before 6 years (*n* = 11) showed no difference in age compared with those with T1D onset after 6 years and controls (*p* = 0.179). Subgroup comparison of P3b markers did not differ (*p* > 0.393).

### 4.2. Glycemic Control

The correlation analysis did not show any relationship between P3b markers (peak time, amplitude, and AUC) and HbA1c (*p* > 0.146) or HbA1c variability (*p* > 0.317); however, exposure score showed a significant relationship with P3b peak time (Pearson *r* = −0.45, *p* = 0.034). We did not find any significant correlation between P3b markers and T1D duration (*p* > 0.130) or age of its onset (*p* > 0.303).

When we grouped T1D patients by insulin regimen (insulin pump, *n* = 12; long-acting human insulins, *n* = 6; combination of short- and long-acting analogs, *n* = 3; human insulin/NPH insulin (excluded from comparison because of the small number of patients), *n* = 1), we found significant (*p* = 0.038) differences in the variability of the P3b peak time. The post hoc *t*-tests did not confirm (*p* = 0.091) shorter P3b latencies for long-acting analogs (366; 352–366 ms) than for insulin pump therapy (388; 383–405 ms) owing to correction for multiple comparisons. P3b amplitude and P3b AUC did not vary among therapies (*p* > 0.09). ANOVA also did not show any significant differences in the variability of P3b markers among patients grouped by T1D complications (*p* > 0.427) or school performance (*p* > 0.463).

In an attempt to reveal any possible dependency of P3b markers on clinical parameters, we conducted multiple linear regression analysis using age of T1D onset, disease duration, and average HbA1c as predictors. The linear models did not show any significant relation (*p* > 0.164).

## 5. Discussion

We compared behavioral performance and electrophysiological brain response during a simple discrimination task between a group of adolescents with T1D and age-matched controls to objectively evaluate the impairment of the executive control suggested by self-reported inventories [22, 23]. We expected a delayed peak time and a lower amplitude for the P3b component or a slower reaction time in adolescents with T1D as similar findings were described formerly [6, 24].

Although the T1D and control groups did not differ significantly in P3b peak time, we observed a trend toward a delayed P3b peak time in the T1D group as found in previous studies [6, 24]. In contrast to our expectations, however, we found that the P3b amplitude was larger in the patients, which confirmed the results of our statistical analysis for the AUC of the P3b component. The present study contrasts with a study by Shehata and Eltayeb [6] describing a drop in N2-P3b amplitude in 40 children (11.7 ± 2.3 years) during an auditory oddball task. We speculate it was likely disease severity that causes this disparity; while 95% of their patients experienced ketoacidosis, the proportion was just 27% among the patients in our study. Shehata and Eltayeb [6] note that the ketoacidosis was a significant factor that negatively correlated with almost all facets of cognitive performance they evaluated. Further, ketoacidosis causes significant morphological and functional brain changes in T1D patients compared to those without ketoacidosis [25].

Similar to our results, Überall et al., studying 29 adolescents with T1D (15.8 ± 3.1 years), did not find any significant difference between them and the control group in the P3b amplitude recorded during visual oddball stimuli [24]; moreover, the grand average of patients' P3b component in their study apparently had a larger AUC than the grand average for controls (see Figure 2 in [24]).

The observed P3b amplitude augmentation in our study is consistent with a recent fMRI work by Gallardo-Moreno et al. [26]. They showed different activation of a brain network involved in a visuospatial working memory task in young adult T1D patients versus controls. Gallardo-Moreno et al. found extended and augmented BOLD signal in the inferior prefrontal cortex, basal ganglia, posterior cerebellum, and substantia nigra. These activation changes occurred without any difference in behavioral performance, consistent with our data and a study by Perantie et al. of young (5–16 years) T1D patients in the go/no-go task [14].

Considering the sensory-processing deficit on the way from the retina up to the primary visual area as indicated by the delayed P100 peak time of the pattern reversal VEPs, we assume that the enhancement of the P3b component reflects compensatory brain activity enabling to achieve a normal behavioral reaction in our T1D subjects.

A similar brain mechanism that can be interpreted as a compensatory response has also been described for adult T1D patients with retinitis in an fMRI study by Wessels et al. [27]. The compensatory neuroplasticity represents a general mechanism described for various pathological (e.g., cognitive load in multiple sclerosis [28]) and physiological (e.g., change detection in aging [29]) conditions involving neural disturbances, and we assume it is the most likely explanation for our results.

Comparing to clinical data, we did not find any relation between the P3b markers and the age of T1D diagnosis (2–16 years), disease duration (2–13 years), or HbA1c (6.5–13.6%), similar to the study of Überall and his colleagues [24].

The used oddball paradigm incorporates a motoric response, which is reflected in prestimulus (readiness potential and negative slope potential) and poststimulus ERP components (motoric potential followed by reafferent sensory response); for review, see [30]. These components might modulate the P3b response. In a further exploration, an evaluation of the motor-related cortical potentials could bring more answers about the compensatory activity we observed.

We emphasize that the selective reaction time and brain responses to oddball stimuli present a response to a rather simple task, and as such, it might not capture the exhaustion and maladaptation of children and adolescents with T1D under the demanding circumstances of T1D treatment and life. The difference between evaluation of EFs by ERPs and multilevel assessment by inventories is worth noting. The second measures broad real-life interplay, taking into account quality of life, treatment adherence, or parental EFs [31]. It shows the frequent failure of EFs in T1D adolescents [4, 32] with a bidirectional consequence for glycemic control and an increased level of T1D-related risk. ERPs analyze EF processing far away from the complexity of life. However, the method reflects, with millisecond accuracy, key elements of executive control such as attention, short-term visual working memory, and decision-making. There is no straightforward relationship between the results of the two approaches, and this situation is not unique, as performance-based measures score different facets of EFs compared with rating measures [33].

## 6. Conclusions

The behavioral performance of adolescents with T1D in a simple oddball test of executive functions was fully comparable with that of the control group; however, the AUC increase of the P3b component suggests a neural mechanism compensating for a subclinical visual impairment manifested by the delayed P100 peak time of pattern-reversal VEPs.

Further research on how adolescents with T1D make their self-management decisions based on EFs should follow two questions: first, whether they have diminished, normal, or advanced functional neurophysiological abilities for such decisions and, additionally, why their life outcomes drop back compared with adolescents without T1D.

## Figures and Tables

**Table 1:** Demographic and diabetes-related characteristics of participating adolescents. Values in the table are expressed as the median and the first and third quartiles.

	T1D patients	Control group	*p*
Number of subjects	22 (10 males, 12 females)	19 (8 males, 11 females)	
Age (years)	15.5 (14.0–16.0)	16.0 (14.5–17.0)	0.231^a^
Visual acuity, logMAR (−)	0.00 (−0.10–0.00)	0.00 (−0.05–0.00)	0.217^a^
Age of T1D diagnosis (years)	8.0 (5.0–10.0)		
Duration of illness (years)	8.0 (5.0–10.8)		
HbA1c IFCC standard (mmol/mol)	68.5 (63.7–76.3)		
HbA1c NGSP (%)	8.4 (8.0–9.1)		
HbA1c coef. variability (%)	7 (5–10)		
Exposure (−)	0.07 (−1.12–0.73)		

^a^Wilcoxon rank-sum test.

**Table 2:** Electrophysiological markers: comparisons between the T1D and control groups. The P3b component recorded in response to the visual oddball test was used to assess executive functions. Listed are the values for the target stimulus evaluated from the parietal derivation (P_Z_–A_2_). The sensory responses from the primary visual cortex were recorded in response to luminance reversal of checkerboard patterns with 40 arcmin and 20 arcmin squares, and peak P100 amplitude and latency were determined in the occipital derivation (O_Z_–A_2_). Values in the table are expressed as the median and the first and third quartiles.

	T1D patients *n* = 22	Control group *n* = 19	*p*
*Responses related to executive functions*			
P3b peak time (ms)	384 (365–396)	368 (348–386)	0.181^b^
P3b amplitude (*μ*V)	27.1 (21.2–33.5)	23.6 (19.7–28.8)	0.331^b^
P3b area under the curve Amplitude × time (*μ*V × ms)	2994 (2541–4047)	2446 (1838–2987)	**0.035 ** ^b^
Reaction time (ms)	354 (333–384)	348 (332–372)	0.713^b^
*The sensory responses of the primary visual cortex*			
R40′ peak time (ms)	107 (105–113)	104 (103–106)	**0.017 ** ^b^
R40′ amplitude (*μ*V)	12.7 (8.6–17.2)	15.7 (12.2–18.9)	0.085^b^
R20′ peak time (ms)	110 (108–114)	108 (105–107)	**0.012 ** ^a^
R20′ amplitude (*μ*V)	10.0 (7.84–16.9)	16.0 (13.0–19.2)	0.066^b^

^a^Wilcoxon rank-sum test, ^b^Student's *t*-test.

## Data Availability

The electrophysiological data used to support the findings of this study are included within the article (see Figure 1). The extracted parameters and clinical measurement data used to support the findings of this study are included within the supplementary information file (available here).

## Abbreviations

ANOVA: Analysis of variance
AUC: Area under the curve
BOLD: Blood oxygenation level-dependent
EFs: Executive functions
ERPs: Event-related potentials
fMRI: Functional magnetic resonance imaging
HbA1c: Hemoglobin A1c
N145: Negative peak of pattern-reversal VEPs
N2: Negative peak of oddball ERPs
N75: Negative peak of pattern-reversal VEPs
P100: Positive peak of pattern-reversal VEPs
P3b: Positive component of the oddball ERPs
PR-VEP 20′: VEPs to pattern reversal of 20 arcmin checkerboard
PR-VEP 40′: VEPs to pattern reversal of 40 arcmin checkerboard
ROC: Receiver operating characteristic
T1D: Type 1 diabetes mellitus
VEPs: Visual evoked potentials.

## Supplementary Materials

Supplementary Materials Table 1: demographic, clinical, behavioral, and electrophysiological data used for statistical evaluation. The first row describes variables as follows: ID: subject identification number; Group: T1D (patients), CON (controls); Sex: 1 (female), 0 (male); Age: age at the time of examination; T1D duration: illness duration; Recorded eye: dominant eye examined; Visual Acuity: fraction 1~4/4 (measured from 4 meters); HbA1c_actual: hemoglobin A1c at time of examination; HbA1c_avg: average hemoglobin A1c; HbA1c_var: variability of hemoglobin A1c; R40_latency: implicit time of dominant peak P100 of pattern-reversal VEPs evoked by reversing chessboard with checks of 40′; R20_latency: similar to preceding but evoked with checks 20′; M20_latency: peak time of dominant peak N2 of motion-onset VEPs evoked by radial motion outside of central 20°; C8_latency: similar to preceding but evoked by motion in central 8°; P300_latency: peak time of dominant peak P300 of ERPs recorded during oddball test; R40_amplitude, R20_amplitude, M20_amplitude, C8_amplitude, and P300_amplitude: amplitudes for appropriate VEP/ERP peaks; P300_auc: area under curve of the peek P300; Reaction_time: reaction time recorded during the oddball test. For details of determining single variables, see Methods. Every row from the third to 43th lists subjects' data.
